# Stereophotogrammetry can feasibly assess ‘physiological’ longitudinal three-dimensional head development of very preterm infants from birth to term

**DOI:** 10.1038/s41598-022-12887-x

**Published:** 2022-05-27

**Authors:** Jana-K. Dieks, Laura Jünemann, Kai O. Hensel, Charlotte Bergmann, Stefan Schmidt, Anja Quast, Sebastian Horn, Matthias Sigler, Philipp Meyer-Marcotty, Petra Santander

**Affiliations:** 1 Department of Paediatric Cardiology, Neonatology and Intensive Care Medicine, University Medical Centre, Robert-Koch-Straße 40, 37075 Göttingen, Germany; 2 Department of Orthodontics, University Medical Centre, Robert-Koch-Straße 40, 37075 Göttingen, Germany; 3 Department of Pediatrics, Faculty of Health, Helios University Medical Center Wuppertal, Witten/Herdecke University, Witten, Germany; 4 Department of Paediatrics, SRH Central Hospital Suhl, Albert-Schweitzer-Straße 2, 98527 Suhl, Germany

**Keywords:** Diseases, Medical research, Risk factors

## Abstract

Head development is a surrogate for brain development in infants and is related to neurocognitive outcome. There is only limited knowledge on early extra-uterine head shape and size assessment in very preterm infants. Here, 26 very preterm infants with a mean gestational age of 29.1 ± 2.2 weeks and a mean birth weight of 1273.8 ± 427.7 g underwent serial stereophotogrammetric 3D head imaging in weekly intervals from birth to term-equivalent age. The main outcome was the longitudinal assessment of the ‘physiological’ preterm head development with cephalometric size (head circumference, cranial volume) and shape parameters (cranial index, cranial vault asymmetry index) according to chronological and postmenstrual age (PMA). Potential clinical risk factors for the development of an abnormal low cranial index (dolichocephaly) were analysed. In serial measurements of 26 infants, the estimated head volume (95% confidence interval) increased from 244 (226–263) cm^3^ at 28 weeks PMA to 705 (688–721) cm^3^ at 40 weeks PMA. Moderate or severe dolichocephaly occurred in 21/26 infants (80.8%). Cranial index decreased over time (72.4%; 70.7–74 95% confidence interval). Brachycephaly and plagiocephaly were uncommon. No risk factors for severe dolichocephaly were identified. Our study shows that early detection of head shape and size anomalies utilizing 3D stereophotogrammetry is feasible and safe even in very preterm infants < 1500 g and/or < 32 weeks. 3D stereophotogrammetry could be used for timely identification of infants at risk for head shape anomalies. No specific risk factors for head shape anomalies were identified, especially not mode and duration of respiratory support.

## Introduction

In preterm infants, optimal early head (and weight) growth determines improved neurodevelopment^1–3^. Likewise, deformational head shape anomalies are known to be associated with suboptimal neurodevelopment^4–6^.

Despite the clinical and scientific significance of head and brain size as well as head shape development, there are still substantial knowledge gaps regarding this topic. Especially preterm infants have a higher risk for insufficient head growth^7^ and various risk factors predispose them for head shape anomalies. Given the fact that being born prematurely is non-physiological in itself, expecting a ‘physiological’ postnatal head development especially in very preterm infants is difficult. Particularly during the first weeks of life, preterm infants more often than term infants have bone mineral deficiency or softer skull. They are earlier exposed to gravitational forces, lateral positioning of the head and respiratory support partly with tight-fitting caps attached to respiratory devices. However, especially in low birthweight infants, head circumference (HC) tape-measurement is limited by suboptimal interobserver reliability^8^.

To assess head shape and volume, three-dimensional (3D) head assessment is favourable, particularly to characterize head shape anomalies in detail. Different radiation-free 3D techniques have been described for this purpose^9,10^ but routine implementation failed due to poor feasibility or cost-effectiveness. Providing a method that allows a bedside, fast and radiation-free image acquisition at an affordable price would enable to routinely identify shape anomalies as well as volumetric measurement of head growth. This could be a window of opportunity for potential preventive interventions, too. To address this, we recently described the feasibility and safety of 3D stereophotogrammetry for head assessment in neonatal patients during hospitalisation^11,12^.

This longitudinal study aims to analyse head shape and size physiology in very preterm infants from birth till term equivalent age using stereophotogrammetry.

## Patients and methods

Between 21/02/2020 and 11/09/2020, consecutive patients were screened for eligibility, prospectively included and underwent serial non-invasive stereophotogrammetric 3D head imaging. Due to institutional regulations regarding the 2020 coronavirus pandemic, inclusion of new patients or image acquisitions were not allowed during the first lockdown in Germany between 16/03/2020 and 21/05/2020. Study inclusion criteria were prematurity with a birth weight < 1500 g and/or gestational age (GA) < 32 weeks. Study exclusion criteria were palliative/comfort care scenarios, inborn neurological disorders, synostotic head shape anomalies, lack of parental consent or expected out-of-hospital patient transfer before term equivalent age. If the first image could not be obtained at 33 weeks PMA at the latest, the patient was excluded from study participation. Exclusion criterion for a single time point image acquisition was any critical or clinically deteriorating health condition.

For each patient, perinatal data was obtained from the medical records. Data of interest included sex, multiple birth status, GA, birth weight, body length and manually measured HC, birth presentation, mode of delivery and presence of asphyxia. At each time of image acquisition, postmenstrual age (PMA), body weight, manually measured HC and details on respiratory support were recorded. Respiratory support was sub classified according to duration of (a) invasive mechanical ventilation (IMV), (b) nasal continuous positive airway pressure (nCPAP) and (c) nasal high-flow therapy (nHFT). For all infants, neonatal morbidity was assessed including death, moderate or severe bronchopulmonary dysplasia (BPD), necrotising enterocolitis (NEC), intracerebral haemorrhage (ICH) ≥ III°, retinopathy of prematurity (ROP) ≥ III° and periventricular leucomalacia (PVL) as well as infection.

Our clinical routine head sonography protocol included repetitive ultrasound assessments of all preterm infants on days 1, 3, 7, 14, and then biweekly until discharge or more frequently in case of abnormal findings at any time.

**Figure 1:**
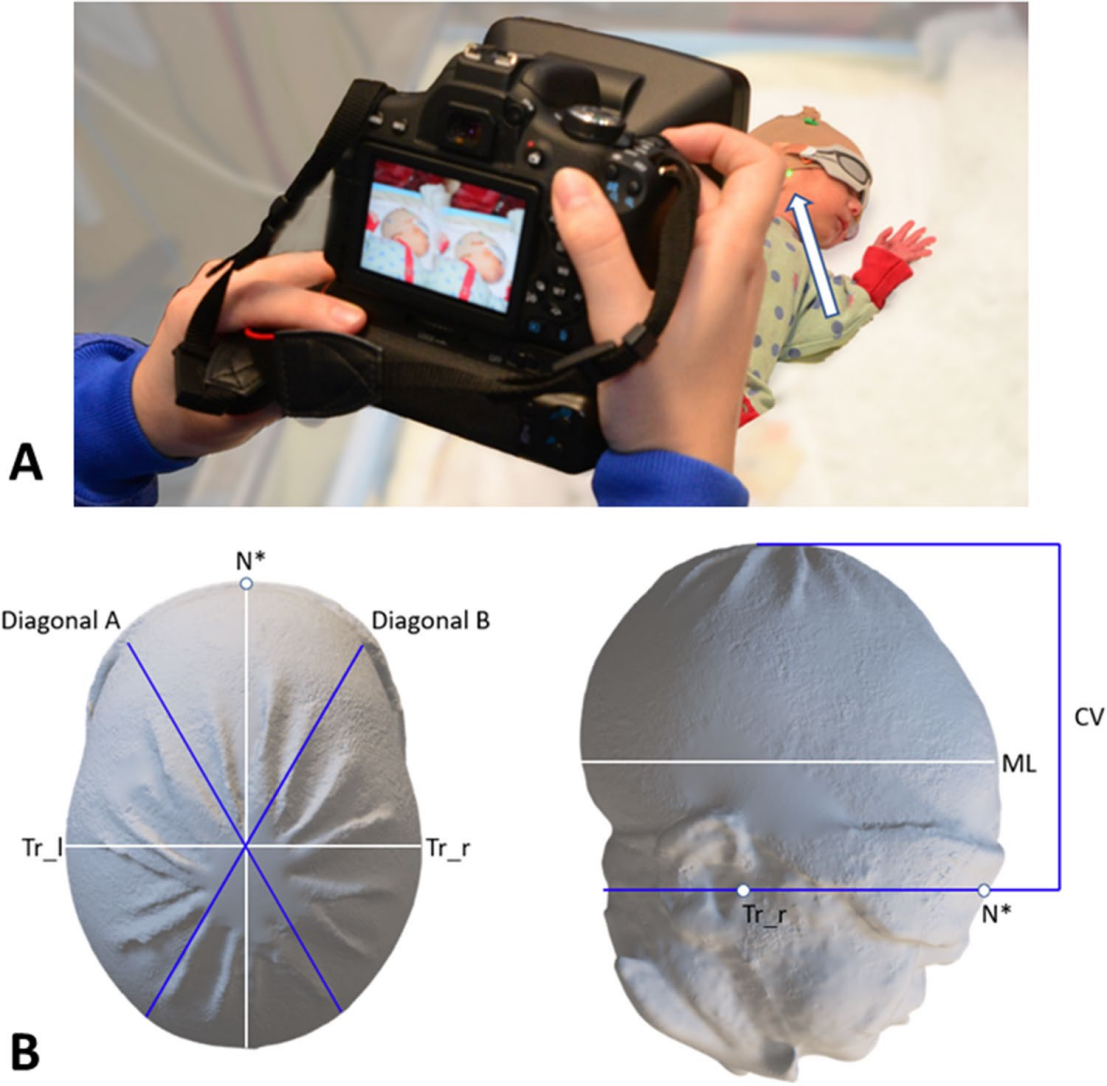
(**A**) Image acquisition. Infant wearing a nylon cap and eye protectors. Arrow points to two merged green light spots that indicate the correct distance (1 m) between the H1 portable camera and the infant’s head. (**B**) Display of the planes and areas for measurements of the infants’ heads. Due to the eye protectors, the anatomic landmark “nasion” is modified and was referred to as nasion* (N*). The nasion*-tragus plane is a free sagittal plane through the nasion*, the left and right tragus (Tr_l/r), respectively. The head level with the maximum anterior–posterior expansion of the head is defined as the measurement level (ML). Head circumference (HC; cm) is the head perimeter around the ML. Cranial volume (CV; m^3^) represents the total head volume cranial to the sagittal plane. Transversal view ((**B**) left) and lateral view ((**B**) right). The cranial index (CI; %) is the proportion of head width to length at measurement level (ML): $CI=\frac{head \,width}{head \,lenght}\times 100.$ The cranial vault asymmetry index (CVAI; %) is the difference between the longest (diagonal A) and shortest 30° diagonal (diagonal B) diameter radiating from the midsagittal plane at ML: $CVAI=\frac{diagonal\, A-diagonal \,B}{major \,diagonal}\times 100.$

For each patient, written informed parental consent was obtained prior to inclusion. In the case of the infant shown in Fig. 1A, specific written informed consent was obtained from the legal guardians that the image can be published in an online open-access publication. The study protocol had been approved by the ethics committee of Georg-August-University Medical Centre (ethics proposal 19/2/18) and is in agreement with the amended Declaration of Helsinki. The study was registered with the German Clinical Trials Register (GermanCTR; ID: DRKS00022938).

### Image acquisition

For image acquisition, a portable stereophotogrammetry Vectra H1 camera (Canfield Scientific, New Jersey, USA) was used. We previously described our neonatal 3D head imaging protocol in detail^11^. In brief, at each session we took ten images from various predefined angles. To reduce hair artifacts, facilitate 3D reconstruction and eye protection, all infants wore smooth nylon caps with textured applications and eye protectors (Neoshades^®^; Kreienbaum Neoscience GmbH, Langenfeld, Rheinland, Germany), respectively. 217 image sets were acquired on the neonatal ward during routine patient care or while the infant was being held in their parents’ arms (Fig. 1A). Images were obtained weekly until discharge or estimated due date, whichever occurred first.

### 3D data analysis and definitions

Images were edited—i.e. processed and converted to a common 3D data format (.stl) using the VECTRA Analysis Module (VAM) software version 6.2.3 (Canfield Scientific, New Jersey, USA) and the MeshLab software version 2016.12 (Visual Computing Lab, ISTI-CNR, Pisa, Italy). Analyses were performed with the Cranioform software version 4.0 (Cranioform, Alpenach Switzerland).

Predefined morphological planes and areas constitute the basis for 3D head measurements (Fig. 1B). Definitions of symmetrical (brachycephaly and dolichocephaly) and asymmetrical head shape anomalies (plagiocephaly) and classification into mild, moderate, and severe according to Wilbrand et al.^13^ and Ifflaender et al.^14^ are presented in Supplemental Table 1.

### Biostatistical analyses

Patient data was handled using Microsoft Excel version 1906 (Redmond, WA USA). Numerical variables are expressed as mean ± standard deviation, categorical variables as percentages. Head parameters with 95% confidence intervals in dependence of age were estimated by polynomial linear regression with random effect patient. Weekly changes of head parameters with 95% confidence intervals were estimated by the same method adjusting for the values of the week before. Manual and digital measurements of HC were compared using Bland–Altman analysis. Associations between HC, cranial volume (CV) and body weight at time of image acquisition were assessed with Pearson correlation coefficient. Associations between dolichocephaly and numerical variables were assessed using the Mann–Whitney U Test. Associations between dolichocephaly and categorical variables were analysed with Fisher’s exact test. Test–retest reliability was assessed with the coefficient of variation. All statistical tests were two-sided with a significance level of 0.05. Statistical analyses were conducted using Stata/IC 16.1 for Unix (StataCorp, 4905 Lakeway Drive College Station, TX 77845, USA).

## Results

Of the 363 patients admitted to our neonatal unit during the study period, 45 met the inclusion criteria. Sixteen patients could not be included due to research restrictions during the first German lockdown in the 2020 coronavirus pandemic. The parents of three patients did not provide consent for study participation. All but four intended images (217 image sets) were acquired at the per protocol predefined time points. The remaining four image sets (at 36 and 37 weeks PMA in a twin couple, respectively) could not be acquired due to coronavirus pandemic-related restrictions. There were no excluded patients.

Of the 26 very preterm infants included, 12 (46.2%) were female and 15 (57.7%) were multiples. Mean GA was 29.1 ± 2.2 weeks. At the time of the first image, the minimal weight was 805 g, and the youngest infant was 27^5^/_7_ weeks PMA.

Mean birth weight was 1273.8 ± 427.7 g (Fenton weight percentile 50.7 ± 22.1^15^) and mean HC was 27.1 ± 3.0 cm. Additional results are reported in Supplemental Text 1.

All included patients received respiratory support with a mean total duration of 34.8 ± 20.9 days. Ten patients (38.5%) were intubated and mechanically ventilated. Overall, mean duration was 1.3 ± 2.9 days for IMV, 15.3 ± 12.7 days for nCPAP, and 18.2 ± 11.9 days for nHFT.

In all patients, vitamin D, phosphorus and calcium levels were monitored and individually substituted to exclude bone mineral deficiency. Positioning regime did not differ within the study cohort and patients were equally put in prone and supine positions.

NEC or PVL were not diagnosed in the here described cohort. Four out of 26 infants (15.4%) experienced in-hospital neonatal morbidity (as defined by above). One infant developed moderate BPD, one infant experienced ICH III° with intraparenchymal haemorrhage and two infants developed ROP ≥ III°. Clinical^16^ or culture-proven sepsis was diagnosed in six infants (23.1%). No study patient developed (post-hemorrhagic) ventriculomegaly or hydrocephalus.

### Head size assessment

**Figure 2:**
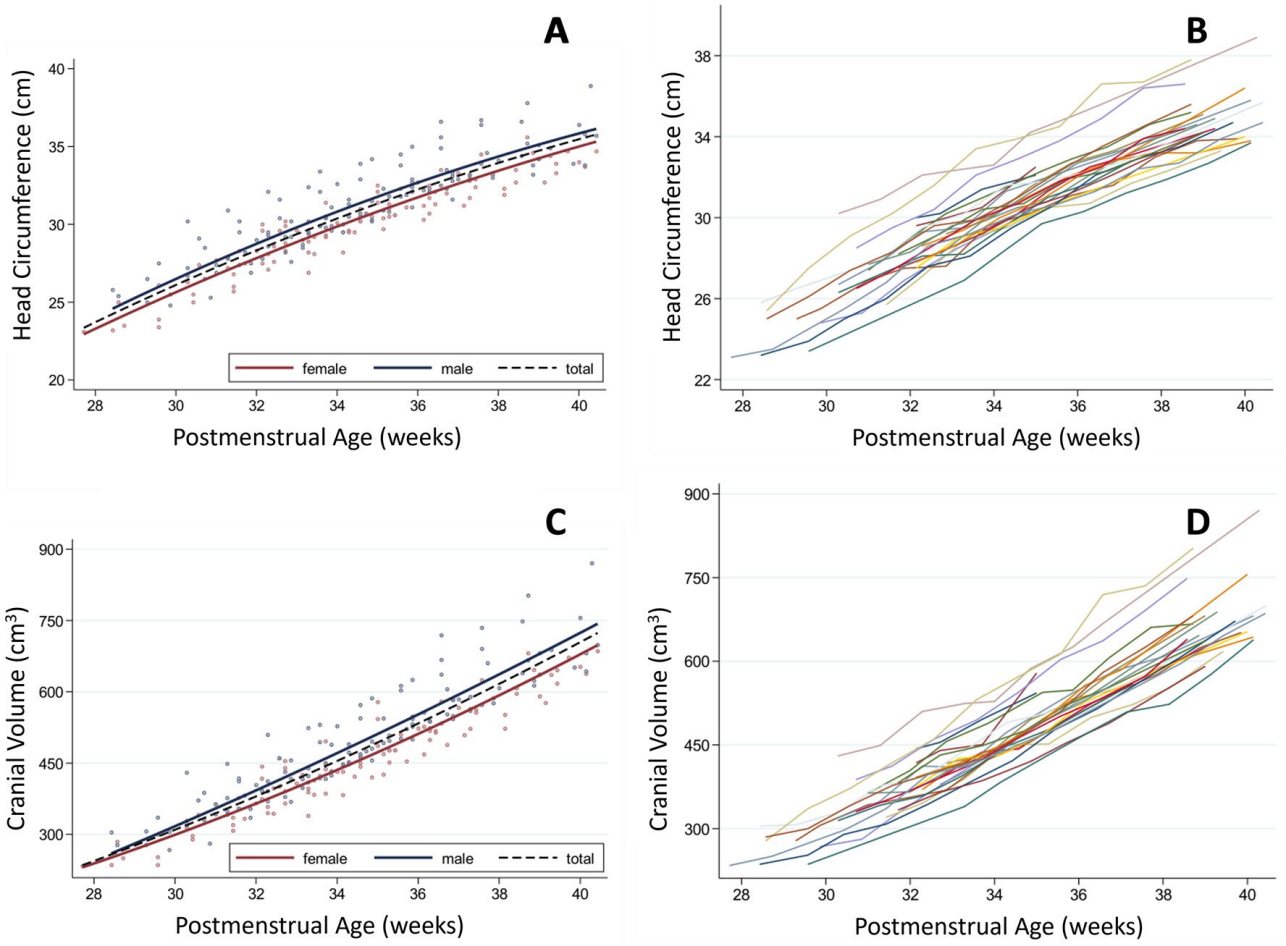
Longitudinal development of head circumference (**A,B**) and cranial volume (**C,D**). Scatter plots of all measurements (n = 217 (**A,C**)) with 2nd degree polynomial regression for all, female and male infants, and individual cranial index graphs (**B,D**).

Numerical digital cephalometric parameters (HC and CV) with 95% confidence intervals according to PMA are displayed in Fig. 2 and Supplemental Table 2. Both female and male infants showed continuous head growth of HC and CV. Weekly HC growth was 1–1.1 cm from 30 to 35 weeks PMA, followed by a gradual decrease of weekly HC growth to 0.6 cm at term equivalent age. Weekly CV gradually increased from 27 to 45 cm^3^ between 30 and 40 weeks PMA.

Manual and digital measurements of HC revealed little deviation. Specifically, the mean difference was − 0.01 (± 0.73) cm with limits of agreement (LOA) − 1.43 to 1.41 cm and a 95% confidence interval of − 0.11 to 0.09 cm (Supplemental Fig. 1).

We detected a strong correlation between HC, CV and body weight, respectively. All pair wise correlation coefficients were > 0.9 (Supplemental Table 3, Supplemental Fig. 2). Correlation between HC, CV and body weight were not influenced by the severity of dolichoceophaly.

### Head shape assessment

**Figure 3:**
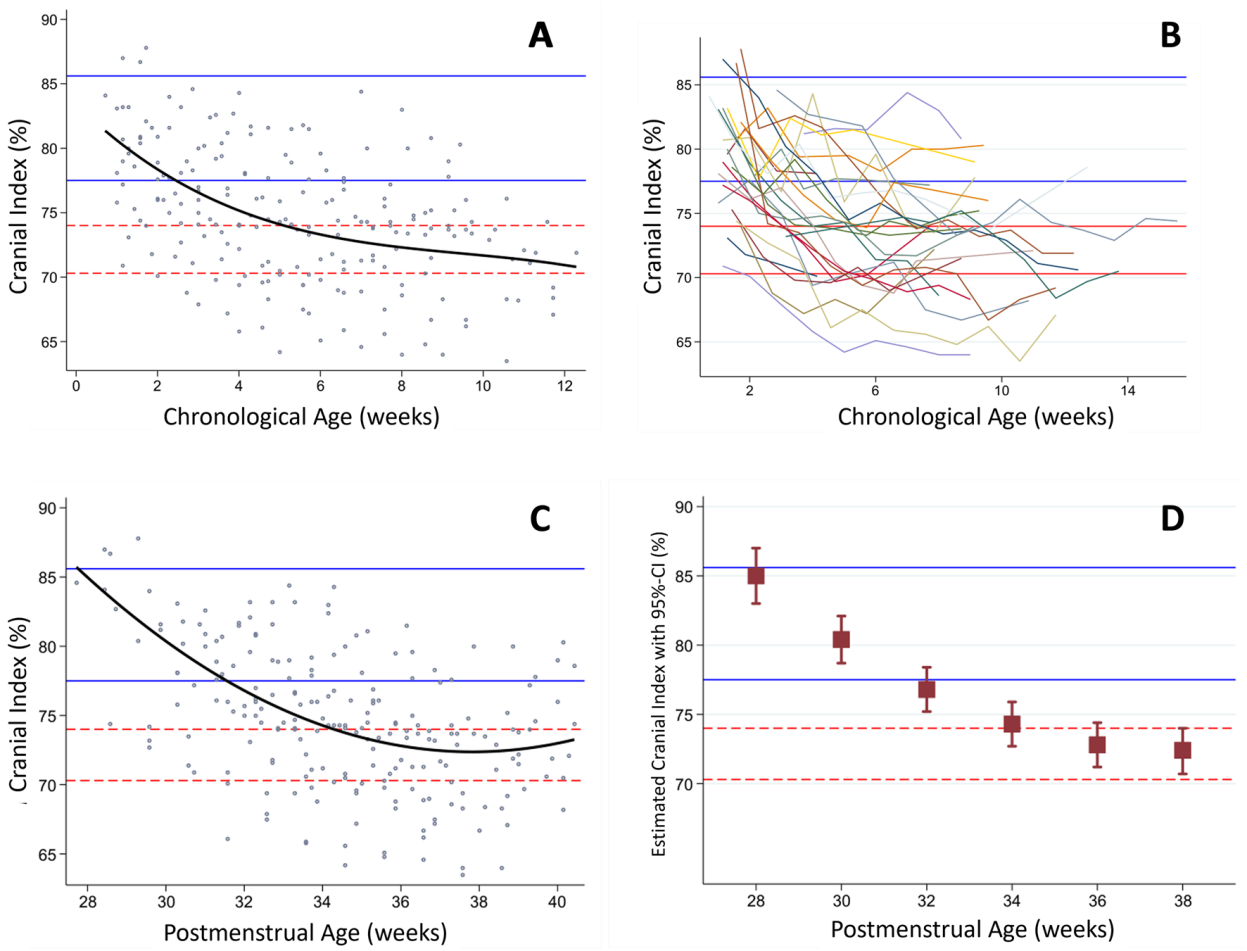
Longitudinal evolvement of cranial index in relation to chronological (**A,B**) and postmenstrual age (**C,D**). Scatter plots of all measurements (n = 217) with 3rd (**A**) and 2nd (**C**) degree polynomial regression, individual cranial index graphs (**B**) and estimation of 2nd‐degree polynomial coefficient with 95% confidence interval (95% CI (**D**)). Mild brachycephaly (above upper blue bar; cranial index 85.6 to 89.5%), normal head morphology (between blue bars; cranial index > 77.5 to < 85.6%), mild dolichocephaly (between lower blue and upper red bar; cranial index 74–77.5%), moderate dolichocephaly (between red bars; cranial index 70.3 to < 74%), severe dolichocephaly (below lower red bar; cranial index < 70.3%). Reference values as defined above^13,14^.

Moderate or severe dolichocephaly was detected in 21/26 infants (80.8%). The CI decreased over time. By 38 weeks PMA at the end of the study period, it reached its nadir with 72.4% and a 95% confidence interval of 70.7–74.0. At discharge, 6/26 infants (23.1%) had normal CI, four (15.4%) had mild, nine (34.6%) moderate and seven (26.9%) severe dolichocephaly. Development of CI and presence of dolichocephaly in relation to chronological age and PMA are illustrated in Fig. 3 and Supplemental Table 4.

Brachycephaly was present in 3/26 infants (13.0%) in the first 2 weeks of age, but never later. These three infants all developed dolichocephaly towards term equivalent age.

Individual CI development for each single patient according to chronological age is presented in Supplemental Fig. 3. The frequency of brachycephaly and dolichocephaly according to chronological age is displayed in Supplemental Table 5.

**Figure 4:**
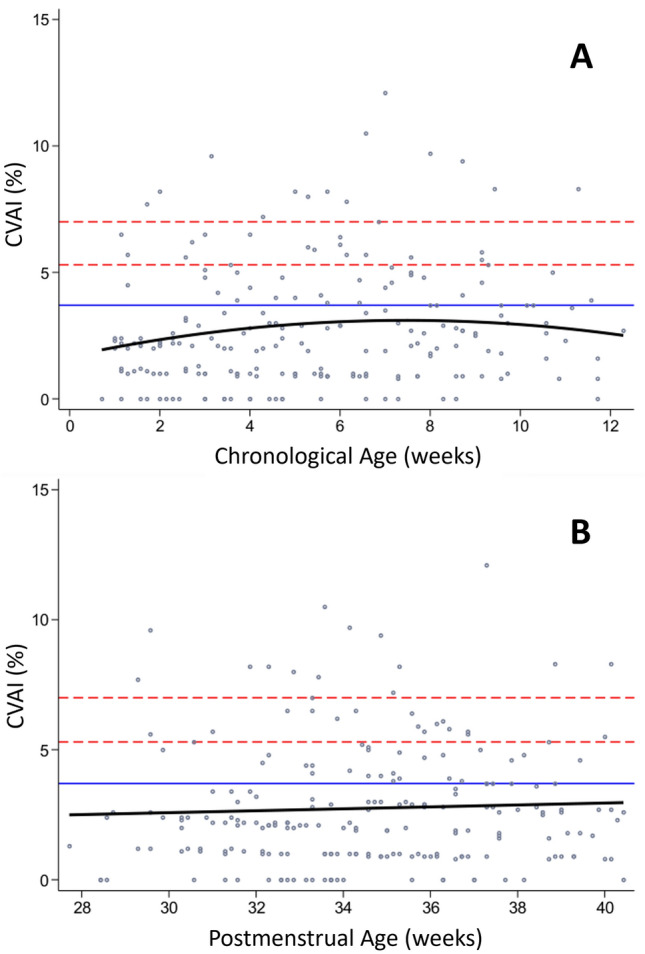
Longitudinal development of plagiocephaly according to chronological (**A**) and postmenstrual age (**B**). Severe plagiocephaly (above upper red bar; cranial vault asymmetry index (CVAI) > 7%), moderate plagiocephaly (between upper and lower red bar; CVAI > 5.3 to 7%), mild plagiocephaly (between lower red and blue bar; CVAI 3.7 to 5.3%), normal cranial vault symmetry (below blue bar; CVAI < 3.7%).

Regarding plagiocephaly, at discharge, 20/26 infants (76.9%) had normal cranial vault asymmetry index (CVAI), three (11.5%) had mild, one (3.8%) had moderate and two (7.7%) had developed severe plagiocephaly. The development of CVAI and presence of plagiocephaly in relation to chronological age and PMA are illustrated in Fig. 4. No association between chronological or PMA and CVAI was observed. Moreover, there was no trend towards an increase of plagiocephaly at term equivalent age.

### Risk factors associated with dolichocephaly

**Figure 5:**
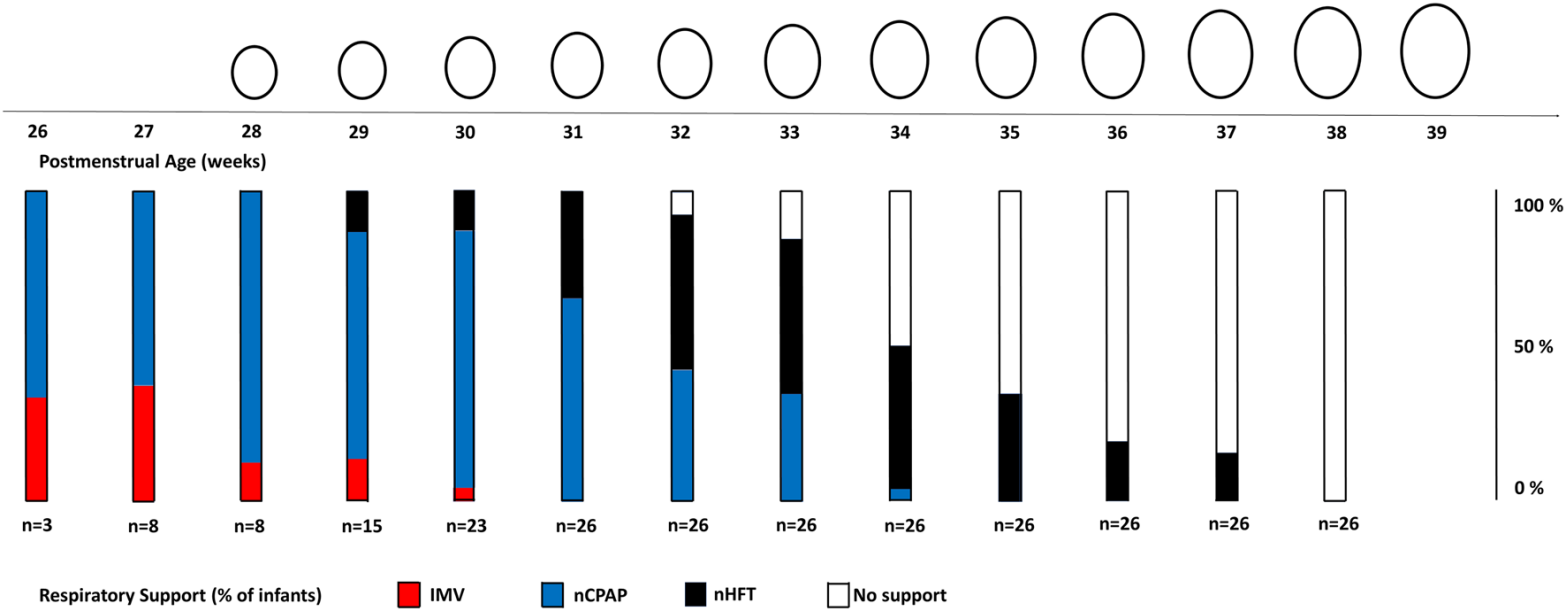
Top: Schematic longitudinal ‘physiological’ head development with mean cranial volume and mean cranial index drawn to scale. Bottom: Total respiratory support subdivided by the different respiratory support modes in relation to postmenstrual age. *IMV* invasive mechanical ventilation; *nCPAP* nasal continuous positive pressure, *nHFT* nasal high-flow therapy.

There were no factors identified to associate with severe dolichocephaly including sex, multiple birth, GA, birth weight, total duration of respiratory support, days of IMV, nCPAP or nHFT, neonatal morbidity, infection, or weight at discharge, respectively (Supplemental Table 6). To further highlight this, Fig. 5 visualizes, that there was no timely correlation between the development of dolichocephaly and mode and duration of respiratory support.

### Test–retest reliability

Test–retest reliability showed a high degree of agreement for cephalometric parameters between repeated measures of one model by one operator (n = 10; Supplemental Table 7).

## Discussion

Head shape and size development is of paramount importance in neonatology. Preterm infants carry an even greater risk for unfavourable head shape and size anomalies^7,14,17,18^ which both independently predict adverse neurodevelopmental outcomes^1–6^.

For the first time, we present data on ‘physiological’ preterm head development longitudinally assessed by 3D stereophotogrammetry in infants as small as < 1500 g and < 32 weeks PMA. This was feasible and safe with no study related adverse events. As a novelty, our study provides normative 3D values for cranial size as well as the symmetrical and asymmetrical head shape parameters cranial index and cranial vault asymmetry index, respectively. Prior to this study the chronological sequence of ‘physiological’ head development in early preterm infancy from 28 weeks PMA to term was poorly described, mainly due to technological shortcomings. For instance, cranial caliper measurement is limited by suboptimal inter-rater re-test reliability^19^. Examining preterm infants as early and accurate as in our current study enables us to identify those at risk for head shape and size anomalies opening a window of opportunity for potential preventive interventions such as an individualized positioning regime.

But what is the ideal parameter best reflecting neurodevelopment in premature infants? Existing growth charts focus on 2D parameters^20^ with HC as the current gold standard. In our present study, we demonstrated that HC correlates with cranial volume. This is in keeping with previous studies^18^. Even the presence of dolichocephaly did not influence head size parameters, which is reassuring for the reliability of this method in clinical routine.

We found that dolichocephaly was frequent in preterm infants, while brachycephaly and plagiocephaly rarely occurred. Until now, scientific research on positional head shape anomalies mainly focused on plagiocephaly and its implications such as long-term neurocognitive impairment^6^. This head shape anomaly occurs later during infancy^17^ which is in keeping with our results. Dolichocephaly in preterm infants between 32 and 34 weeks PMA was associated with adverse motor outcomes^21^. For neurocognitive development, however, to the best of our knowledge, an association with dolichocephaly still needs to be determined. To address this knowledge gap, there is need for further studies, specifically for long-term neurologic outcome after head shape anomalies identified during the neonatal period—including both plagiocephaly and dolichocephaly. Thereby, the potential of early head morphology as a prognostic marker for neurocognitive development can be determined. It is also of great interest, if the severity of early dolichocephaly is related to a greater risk of the subsequent development of plagiocephaly and thereby may serve as a prognostic marker. Assuming a relationship between head molding and clinical outcome, a routine bedside head shape and size assessment in early preterm infancy could facilitate preventive and therapeutic options. 3D stereophotogrammetry to monitor head development in preterm infancy seems promising as it fulfils several important requirements of a routine measure in neonatal care: The application time during infant care is short, and image acquisition is radiation-free and inexpensive.

In our study, no underlying potential risk factors were identified that may account for the development of dolichocephaly. Interestingly, thereby our data does not support the widespread assumption that respiratory support and especially nCPAP treatment is a risk factor for dolichocephaly. Evidence regarding this is sparse and data focusing on the development of dolichocephaly is lacking. However, in contrast to our study results, Ifflaender and colleagues demonstrated the median duration of total respiratory support, nCPAP and IMV, respectively, to be significantly longer in dolichocephalic infants compared to non-dolichocephalic controls^14^. In line with our findings, McCarty et al. did not identify BPD—which most often occurs after long-term ventilation—or any other neonatal morbidities to be associated with dolichocephaly.

One study patient developed high grade ICH, which was closely monitored by regular head ultrasound assessments and head circumference measurements. Comparing the 3D parameters to the rest of the here described cohort, we found that the patient developed microcephaly and severe plagiocephaly (CVAI max. 9.7%), but no dolichocephaly (CI min. 80.8%). The patient was not excluded from analysis because there was no evidence for post-hemorrhagic ventriculomegaly or hydrocephalus and to acknowledge ICH as a well-known complication of preterm infancy.

### Limitations

Although still significant with regards to the special patient population, the number of study participants was relatively low. While the sample size was sufficient to demonstrate feasibility and safety of the new procedure, this may explain the surprising missing detection of potential underlying risk factors for dolichocephaly. As we used a novel technology, we pragmatically chose a single-centre study design and included consecutive patients to avoid selection bias. Unfortunately, we were not able to include as many patients as initially planned because the study conduction period fell in the time of the *first wave* of the coronavirus pandemic in early 2020—a time in which clinical research has been severely disrupted worldwide^22^.

Following the predefined in- and exclusion criteria, all measurements were included into the analysis although one study patient developed high grade ICH which despite the absence of post-hemorrhagic hydrocephalus possibly influences the head shape and size development.

Moreover, the here selected study design does not feature a control group. The reason was the nature of the studied phenomenon, in other words, there is no standard reference for extrauterine head development in very preterm infants.

Finally, like with many novel technologies, data handling (i.e. 3D image reconstruction and subsequent analyses) is not yet fully automated for image sets acquired by hand-held cameras. This results in a somewhat cumbersome workflow rendering the method less likely to be clinically implemented at this point. A solution would be an automated image processing tool, analogue to the technology available for stationary 3D stereophotogrammetry systems.

## Conclusion

Using 3D stereophotogrammetry, evaluation of ‘physiological’ head shape and size development was feasible and safe even in very preterm infants < 1500 g and a PMA < 32 weeks. Being fast, accurate, radiation-free and inexpensive, 3D stereophotogrammetry seems suitable as a routine measure in neonatal care to assess head shape and size in infants at risk.

## Supplementary Information

Supplementary Information.
