# Supplementary material for: Stereophotogrammetry can feasibly assess ‘physiological’ longitudinal three-dimensional head development of very preterm infants from birth to term
Source: Sci Rep. 2022 May 27;12:8940. doi: 10.1038/s41598-022-12887-x (PMC9136805; doi:10.1038/s41598-022-12887-x)
Supplement: Supplementary file 1 — Supplementary Information. [file 41598_2022_12887_MOESM1_ESM.docx]

**Supplement to “Stereophotogrammetry can feasibly assess ‘physiological’ longitudinal three-dimensional head development of very preterm infants from birth to term”**

**Supplemental text 1**

Ethnicity was Caucasian European (n=22; 84.6%), Pacific (n=1; 3.8%), African-American (n=2; 7.7%), and Arabian (n=1; 3.8%), respectively. Birth presentation was cephalic position in 16 (64.0%). The delivery mode was caesarean section in 23 infants (88.5%). 5-/10-minute Apgar scores were 5/7 or higher in all patients. Range of umbilical cord arterial pH was between 7.24 and 7.35. Mean CRIB (clinical risk index for babies) score was 5.0±3.6.

**Supplemental figures**


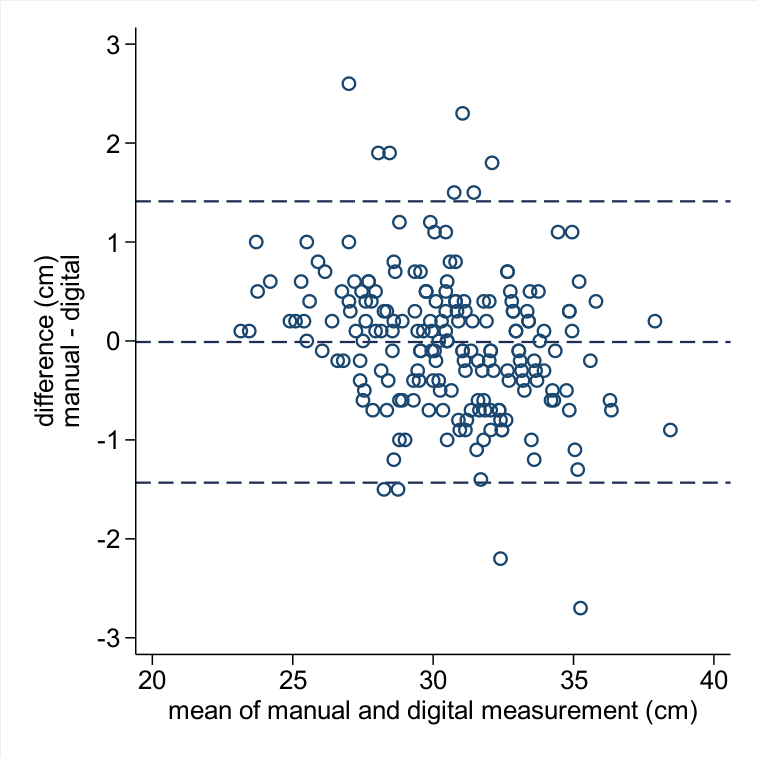


***Supplemental figure 1:*** Bland-Altman Plot of manual versus digital head circumference.


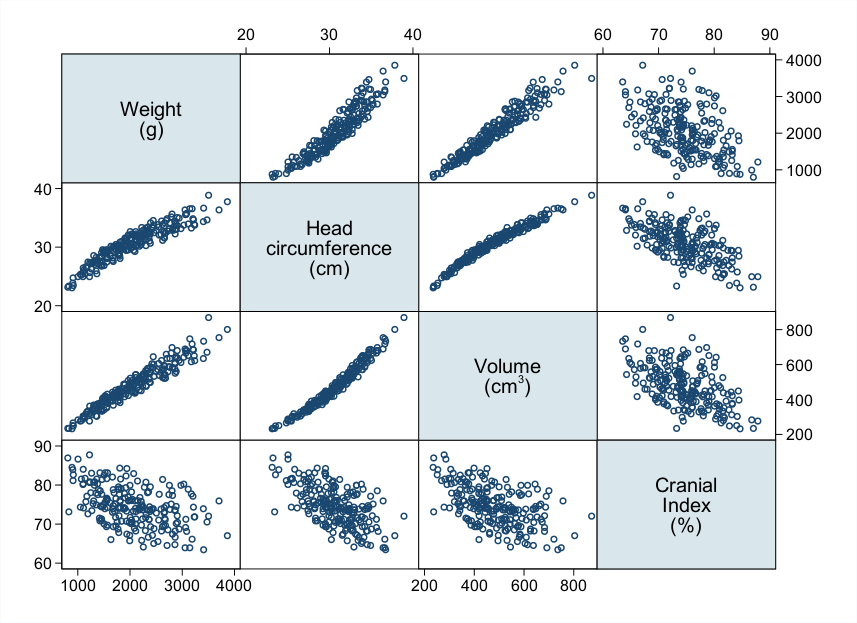


***Supplemental figure 2:*** Scatterplot matrix for body weight, head circumference (HC), cranial volume (CV) and cranial index (CI).


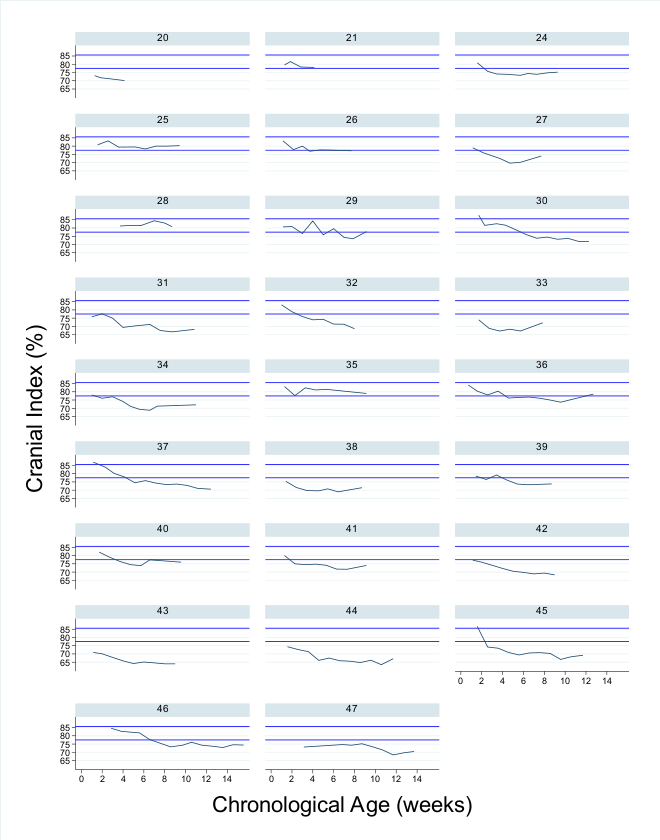


***Supplemental figure 3***: Individual CI development for each single patient according to chronological age.

**Supplemental tables**

***Supplemental table 1:*** Definitions of symmetrical and asymmetrical non-synostotic head shape anomalies and classification into mild, moderate and severe. Reference values according to Wilbrand et al. [[1](#_ENREF_1)] and Ifflaender et al. [[2](#_ENREF_2)].

|  | **Symmetry of  head shape anomaly** | **mild** | **moderate** | **severe** |
| --- | --- | --- | --- | --- |
| Brachycephaly | symmetrical | CI = 75th to 90th percentile:  85.6 to 89.5% | CI = >90th to 97th percentile:  >89.5 to 94.2% | CI >97th percentile:  >94.2% |
| Dolichocephaly | symmetrical | CI = 10th to 25th percentile:  74 to 77.5% | CI = 3rd to <10th percentile:  70.3 to <74% | CI <3rd percentile:  <70.3% |
| Plagiocephaly | asymmetrical | CVAI = 75th to 90th percentile: 3.7 to 5.3% | CVAI = >90th to 97th percentile: >5.3 to 7% | CVAI >97th percentile: >7% |

CI = cranial index; CVAI = cranial vault asymmetry index.

***Supplemental table 2:*** Longitudinal development of estimated head circumference and cranial volume for all, female, and male infants according to postmenstrual age (n=217).

|  | **All infants**  **n=26** | **Female**  **n=12** | **Male**  **n=14** |
| --- | --- | --- | --- |
| ***Postmenstrual Age*** | ***Head circumference (cm; 95% confidence interval)*** | | |
| *28* | 23.7 (23.2-24.2) | 23.2 (22.5-23.9) | 24.1 (23.5-24.8) |
| *29* | 24.9 (24.5-25.4) | 24.4 (23.8-25.1) | 25.4 (24.7-26.0) |
| *30* | 26.1 (25.7-26.6) | 25.6 (25.0-26.2) | 26.5 (25.9-27.1) |
| *31* | 27.2 (26.8-27.7) | 26.7 (26.1-27.4) | 27.7 (27.1-28.2) |
| *32* | 28.3 (27.9-28.8) | 27.8 (27.2-28.5) | 28.7 (28.2-29.3) |
| *33* | 29.4 (29.0-29.8) | 28.9 (28.3-29.5) | 29.8 (29.2-30.4) |
| *34* | 30.4 (30.0-30.8) | 29.9 (29.3-30.5) | 30.8 (30.2-31.4) |
| *35* | 31.3 (30.9-31.8) | 30.8 (30.2-31.5) | 31.7 (31.2-32.3) |
| *36* | 32.3 (31.8-32.7) | 31.8 (31.1-32.4) | 32.7 (32.1-33.2) |
| *37* | 33.1 (32.7-33.5) | 32.6 (32.0-33.2) | 33.5 (33.0-34.1) |
| *38* | 34.0 (33.5-34.4) | 33.4 (32.8-34.1) | 34.4 (33.8-34.9) |
| *39* | 34.7 (34.3-35.2) | 34.2 (33.6-34.9) | 35.1 (34.6-35.7) |
| *40* | 35.5 (35.0-35.9) | 35.0 (34.3-35.6) | 35.9 (35.3-36.5) |
|  | ***Head volume (cm^3^; 95% confidence interval)*** | | |
| 28 | 244 (226-263) | 225 (201-250) | 260 (237-283) |
| 29 | 277 (260-293) | 258 (235-280) | 292 (271-314) |
| 30 | 310 (295-326) | 291 (269-313) | 326 (306-346) |
| 31 | 345 (330-360) | 326 (304-347) | 360 (341-380) |
| 32 | 380 (366-395) | 361 (340-383) | 396 (376-416) |
| 33 | 417 (403-432) | 398 (377-419) | 433 (413-452) |
| 34 | 455 (440-469) | 436 (414-457) | 471 (451-490) |
| 35 | 494 (479-508) | 475 (453-496) | 509 (490-529) |
| 36 | 534 (519-548) | 515 (493-536) | 549 (530-569) |
| 37 | 575 (560-589) | 556 (534-577) | 591 (571-610) |
| 38 | 617 (602-632) | 598 (576-619) | 633 (613-653) |
| 39 | 660 (645-676) | 641 (619-663) | 676 (656-696) |
| 40 | 705 (688-721) | 685 (663-708) | 720 (699-741) |

***Supplemental table 3:*** Pearson correlation of HC, CV and body weight.

|  | **Cranial volume and head circumference** | **Cranial volume and body weight** | **Body weight and head circumference** | **Body weight and cranial index** | **Cranial volume and cranial index** |
| --- | --- | --- | --- | --- | --- |
| ***All images***  ***(n = 217)*** | 0.981 | 0.962 | 0.934 | -0.472 | -0.532 |
| ***No/mild dolichocephaly***  ***(n = 117)*** | 0.982 | 0.966 | 0.935 | -0.328 | -0.383 |
| ***Moderate/severe dolichocephaly (n = 100)*** | 0.977 | 0.942 | 0.916 | -0.247 | -0.300 |

***Supplemental table 4A***: Estimated cranial index and 95% confidence intervals according to chronological age (third degree polynomial linear regression).

| **Chronological age (weeks)** | **Cranial index (%)** | **95% confidence interval** |
| --- | --- | --- |
| 2 | 78.4 | 76.7-80.0 |
| 4 | 75.2 | 73.5-76.8 |
| 6 | 73.3 | 71.7-75.0 |
| 8 | 72.3 | 70.6-74.0 |
| 10 | 71.7 | 69.9-73.4 |
| 12 | 70.9 | 69.1-72.8 |

***Supplemental table 4B***: Estimated cranial index and 95% confidence intervals according to postmenstrual age (second degree polynomial linear regression).

| **Postmenstrual age (weeks)** | **Cranial index (%)** | **95% confidence interval** |
| --- | --- | --- |
| 28 | 85.0 | 83.0-87.0 |
| 30 | 80.4 | 78.7-82.1 |
| 32 | 76.8 | 75.2-78.4 |
| 34 | 74.3 | 72.7-75.9 |
| 36 | 72.8 | 71.2-74.4 |
| 38 | 72.4 | 70.7-74.0 |

***Supplemental table 5:*** Frequency of brachycephaly and dolichocephaly according to chronological age, number of infants (%).

| **Chronological** | **Infants** | **brachycephaly** | **normal** | **dolichocephaly** | | |
| --- | --- | --- | --- | --- | --- | --- |
| **age (weeks)** | **(n)** | **mild** | **Cranial index** | **mild** | **moderate** | **severe** |
| < 2 | 23 | 3 (13) | 13 (56.5) | 4 (17.4) | 3 (13.0) | 0 (0) |
| >= 2 | 26 | 0 (0) | 8 (30.8) | 10 (38.5) | 5 (19.2) | 3 (11.5) |
| >= 4 | 25 | 0 (0) | 7 (28.0) | 4 (16.0) | 5 (20.0) | 9 (36.0) |
| >= 6 | 23 | 0 (0) | 2 (8.7) | 6 (26.1) | 9 (39.1) | 6 (26.1) |
| >= 8 | 20 | 0 (0) | 4 (20.0) | 2 (10.0) | 8 (40.0) | 6 (30.0) |
| >= 10 | 8 | 0 (0) | 0 (0) | 1 (12.5) | 3 (37.5) | 4 (50.0) |
| >= 12 | 5 | 0 (0) | 1 (20.0) | 0 (0) | 3 (60.0) | 1 (20.0) |

* in infants with multiple measurements within a time period, the lowest cranial index within this time period was used.

***Supplemental table 6A***: Severe dolichocephaly at discharge – categorical variables.

|  |  | **Total (n)** | **No** | **Yes** | **OR (95%-CI)** | ***p*-value*** |
| --- | --- | --- | --- | --- | --- | --- |
| **Sex** | female | 12 | 9 (75.0%) | 3 (25.0%) |  |  |
|  | male | 14 | 10 (71.4%) | 4 (28.6%) | 1.20 (0.20-7.14) | 1.000 |
| **Birth weight in** | < 1000 | 6 | 4 (66.7%) | 2 (33.3%) |  |  |
| **categories [g]** | 1000-1500 | 14 | 10 (71.4%) | 4 (28.6%) |  |  |
|  | >1500 | 6 | 5 (83.3%) | 1 (16.7%) |  | 1.000 |
| **Multiple birth** | no | 15 | 13 (86.7%) | 2 (13.3%) |  |  |
|  | yes | 11 | 6 (54.5%) | 5 (45.5%) | 5.42 (0.68-43.32) | 0.095 |
| **Complications**** | no | 22 | 15 (68.2%) | 7 (31.8%) |  |  |
|  | yes | 4 | 4 (100%) | 0 (0%) |  | 0.546 |
| **Infection** | no | 20 | 14 (70.0%) | 6 (30.0%) |  |  |
|  | yes | 6 | 5 (83.3%) | 1 (16.7%) | 0.47 (0.04-5.23) | 1.000 |

* Fisher's exact test; ** one or more of the following: death, moderate or severe bronchopulmonary dysplasia (BPD), necrotizing enterocolitis (NEC), intracerebral hemorrhage (ICH) ≥III°, retinopathy of prematurity (ROP) ≥III° and periventricular leucomalacia (PVL); OR (95%-CI) = odds ratio with 95% confidence intervals.

***Supplemental table 6B***: Severe dolichocephaly at discharge – numerical variables.

|  | **Dolichoco-cephaly** | **n** | **Mean** | **Sd** | **Median** | **IQR** | **Min.-Max.** | ***p*-value*  OR (95-CI)** |
| --- | --- | --- | --- | --- | --- | --- | --- | --- |
| **Gestational age** | No | 19 | 29.3 | 2.0 | 30.3 | 27.6-30.9 | 24.9-31.6 | 0.339 |
| **[weeks]** | Yes | 7 | 28.9 | 1.4 | 29.3 | 27.0-29.6 | 27.0-30.9 | 0.90 (0.57-1.44) |
| **Birth weight [kg]** | No | 19 | 1.3 | 0.4 | 1.2 | 1.1-1.6 | 0.6-1.8 | 0.885 |
|  | Yes | 7 | 1.3 | 0.3 | 1.4 | 1.0-1.5 | 0.9-1.8 | 1.39 (0.11-17.62) |
| **nCPAP [d]** | No | 19 | 3.0 | 0.4 | 3.0 | 2.8-3.2 | 2.3-3.7 | 0.751 |
| **At onset of dolichoc.** | Yes | 7 | 3.0 | 0.5 | 3.1 | 2.7-3.1 | 2.1-3.9 | 0.90 (0.12-6.90) |
| **Total resp. support [d]** | No | 19 | 17.8 | 14.1 | 10.0 | 7.0-27.0 | 5.0-46.0 | 0.270 |
| **At onset of dolichoc.** | Yes | 7 | 8.7 | 2.8 | 10.0 | 6.0-10.0 | 5.0-13.0 | 0.95 (0.88-1.01) |
| **Invasive ventilation [d]** | No | 19 | 36.0 | 23.0 | 29.0 | 16.0-61.0 | 7.0-79.0 | 0.908 |
| **At onset of dolichoc.** | Yes | 7 | 31.6 | 14.4 | 32.0 | 27.0-35.0 | 7.0-56.0 | 0.99 (0.95-1.03) |
| **Nasal high-flow [d]** | No | 19 | 1.7 | 3.3 | 0.0 | 0.0-3.0 | 0.0-14.0 | 0.321 |
| **At onset of dolichoc.** | Yes | 7 | 0.3 | 0.5 | 0.0 | 0.0-1.0 | 0.0-1.0 | 0.85 (0.63-1.14) |
| **Gestational age** | No | 19 | 16.5 | 11.6 | 16.0 | 11.0-24.0 | 0.0-37.0 | 0.163 |
| **[weeks]** | Yes | 7 | 22.6 | 12.8 | 24.0 | 17.0-26.0 | 0.0-43.0 | 1.04 (0.97-1.12) |

* Mann-Whitney-U-Test; IQR = interquartile range; OR (95%-CI) = odds ratio with 95% confidence intervals; Sd = standard deviation.

**Test-retest reliability**

***Supplemental table 7***: Repeated measures of one model by one operator (n=10).

|  | **Minimum-Maximum** | **Mean (standard deviation)** | **Coefficient of variation** | **95% confidence interval of mean** | **rho, *p*-value** |
| --- | --- | --- | --- | --- | --- |
| **Head circumference [cm]** | 31.9-32.0 | 31.9 (0.0) | 0.00 | 31.9-32.0 | 0.19, p=0.599 |
| **Cranial index** | 82.6-83.3 | 83.2 (0.3) | 0.00 | 82.9-83.4 | 0.17, p=0.631 |
| **Vertex height** | 7.9-8.1 | 8.0 (0.1) | 0.01 | 8.0-8.1 | -0.41, p=0.241 |
| **30° diagonal Δ** | 0.10-0.20 | 0.14 (0.05) | 0.37 | 0.10-0.18 | 0.71, p=0.021 |
| **30°cranial vault asymmetry index** | 1.0-1.9 | 1.4 (0.5) | 0.34 | 1.0-1.7 | 0.71, p=0.021 |
| **Cranial volume [cm^3^]** | 482.1-488.5 | 485.5 (2.1) | 0.00 | 484.0-487.0 | -0.73, p=0.016 |
| **Posterior vault asymmetry index** | 0.2-6.3 | 3.1 (2.1) | 0.69 | 1.6-4.6 | 0.49, p=0.148 |
| **Ear Offset [mm]** | 0.10-0.20 | 0.12 (0.04) | 0.35 | 0.09-0.15 | -0.26, p=0.466 |
| **Vertical Ear Offset [mm]** | 0.00-0.40 | 0.21 (0.14) | 0.69 | 0.11-0.31 | -0.50, p=0.140 |

**References**

1. Wilbrand JF, Schmidtberg K, Bierther U, et al. Clinical classification of infant nonsynostotic cranial deformity. The Journal of pediatrics 2012;**161**(6):1120-5 doi: 10.1016/j.jpeds.2012.05.023[published Online First: Epub Date]|.

2. Ifflaender S, Rudiger M, Konstantelos D, Wahls K, Burkhardt W. Prevalence of head deformities in preterm infants at term equivalent age. Early human development 2013;**89**(12):1041-7 doi: 10.1016/j.earlhumdev.2013.08.011[published Online First: Epub Date]|.
